# Neuroimaging in the Rare Sleep Disorder of Kleine–Levin Syndrome: A Systematic Review

**DOI:** 10.3390/clockssleep4020025

**Published:** 2022-05-31

**Authors:** Juan Fernando Ortiz, Jennifer M. Argudo, Mario Yépez, Juan Andrés Moncayo, Hyder Tamton, Alex S. Aguirre, Ghanshyam Patel, Meghdeep Sen, Ayushi Mistry, Ray Yuen, Ahmed Eissa-Garces, Diego Ojeda, Samir Ruxmohan

**Affiliations:** 1 California Institute of Behavioral Neuroscience & Psychology, Fairfield, CA 94534, USA; 2 Faculty of Medical Sciences, School of Medicine, Universidad de Cuenca, Cuenca 010107, Ecuador; argudojennifer@gmail.com; 3 School of Medicine, Universidad Católica Santiago de Guayaquil, Guayaquil 090615, Ecuador; marioayepez@gmail.com; 4 Department of Neurology, Pontificia Universidad Católica del Ecuador Quito, Quito 170143, Ecuador; jmoncayo725@gmail.com; 5 Neurology Department, Larkin Community Hospital, South Miami, FL 33143, USA; htamton@gmail.com (H.T.); ryuen@auis.edu (R.Y.); ruxmohan@yahoo.com (S.R.); 6 School of Medicine, Colegio de Ciencias de la Salud, Universidad San Francisco de Quito, Quito 170901, Ecuador; aguaxindeclaus@gmail.com (A.S.A.); aeissag@estud.usfq.edu.ec (A.E.-G.); dojeda@gmail.com (D.O.); 7 Mercy Health Internal Medicine Residency, Javon Bea Hospital, Rockford, IL 61114, USA; grp_aaa@yahoo.com; 8 School of Medicine, American University of Antigua, Osbourn, Antigua and Barbuda; meghdeepsen@gmail.com; 9 Pramukh Swami Medical College, Karamsad 388325, Gujarat, India; ayushimistry@gmail.com

**Keywords:** Kleine–Levin syndrome, SPECT, P.E.T., fMRI, hypersexuality, apathy, derealization

## Abstract

Kleine–Levin syndrome (KLS) is characterized by episodes of hypersomnia. Additionally, these patients can present with hyperphagia, hypersexuality, abnormal behavior, and cognitive dysfunction. Functional neuroimaging studies such as fMRI-BOLD, Positron Emission Tomography (PET) or SPECT help us understand the neuropathological bases of different disorders. We conducted a systematic review to investigate the neuroimaging features of KLS patients and their clinical correlations. This systematic review was conducted by following the Meta-Analysis of Observational Studies in Epidemiology (MOOSE) and PRISMA protocol reporting guidelines. We aim to investigate the clinical correlation with neuroimaging among patients with KLS. We included only studies written in the English language in the last 20 years, conducted on humans; 10 studies were included. We excluded systematic reviews, metanalysis, and case reports. We found that there are changes in functional imaging studies during the symptomatic and asymptomatic periods as well as in between episodes in patients with K.L.S. The areas most reported as affected were the hypothalamic and thalamic regions, which showed hypoperfusion and, in a few cases, hyperperfusion; areas such as the frontal, parietal, occipital and the prefrontal cortex all showed alterations in cerebral perfusion. These changes in cerebral blood flow and regions vary according to the imaging (SPECT, PET SCAN, or fMRI) and the task performed while imaging was performed. We encountered conflicting data between studies. Hyper insomnia, the main feature of this disease during the symptomatic periods, was associated with decreased thalamic activity. Other features of K.L.S., such as apathy, hypersexuality, and depersonalization, were also correlated with functional imaging changes. There were also findings that correlated with working memory deficits seen in this stage during the asymptomatic periods. Hyperactivity of the thalamus and hypothalamus were the main features shown during the asymptomatic period. Additionally, functional imaging tends to improve with a longer course of the disease, which suggests that K.L.S. patients outgrow the disease. These findings should caution physicians when analyzing and correlating neuroimaging findings with the disease.

## 1. Introduction

The first cases of Kleine–Levin syndrome (K.L.S.) were described by Kleine and Levin separately, in 1925 and 1929, respectively [1]. Even though cases of K.L.S. were reported worldwide, according to studies published, it was more frequent among young adolescent Caucasians, as established by a study in Israel [2], and in those of Ashkenazi Jews in Western countries [3]. K.L.S. has an estimated prevalence of 1–5 cases in a million, with males being more affected, at a proportion of 60–87% [4]. Most of these cases are sporadic, but familial cases have also been reported [4]. 

The disorder is characterized by episodic hypersomnia; each episode lasts ten days and recurs every 3.5 months on average [4]. Patients usually outgrow the disease after eight years [4]. The disorder also presents with cognitive and behavioral abnormalities, such as confusion, agitation, derealization, etc. [4].

The cases also report normal periods of cognition and behavior in between these episodes [5]. A study by Arnulf et al. describes episodes of abnormal cognition and hypersomnia, lasting anywhere between 2.5 and 80 days, with the duration between each episode ranging from 15 days to 6 years [3]. Other symptoms which were reported included eating disorders (hyperphagia), disinhibited behavior (hypersexuality), and affect changes (depressed mood, anxiety) [5]. This syndrome is seen to be distinctively different in males and females, with women reporting a longer duration of the disease, higher frequency of affect changes, and lower hypersexuality frequency [6]. 

The etiology of this syndrome is not well understood. It is hypothesized that hypothalamic pathology plays a role in the development of the syndrome, but an exact association has not been made [6]. Other possible triggers include infection, trauma, toxins, neurotransmitter abnormalities, autoimmune dysfunction, and psychological disturbances [7].

This syndrome is diagnosed using the International Classification of Sleep Disorders. Table 1 shows the criteria for K.L.S. [7].

Radiological investigations conducted in cases of K.L.S. were mostly to identify and treat potential causes such as meningitis or encephalitis (CSF), focal brain lesions (brain imaging), or epilepsy (E.E.G.) [8].

Among all the treatment modalities available, no definitive treatment has been found. In one study, 41% of patients showed termination of episodes and a “clear mind” with amantadine, an antiviral with dopamine reuptake inhibitory capacity [6]. Their study showed that lithium has the highest response rate to other medical interventions. However, Oliveira M et al. conducted a search for trials in the Cochrane database but could not find any substantial evidence for the use of pharmacological therapies such as benzodiazepines, lithium, stimulants, anti-depressants, anti-psychotics, anti-epileptics or others [9].

## 2. Methods

### 2.1. Protocol

This systematic review was conducted by following the Meta-Analysis of Observational Studies in Epidemiology (MOOSE) and PRISMA protocol reporting guidelines [10,11].

#### 2.1.1. Eligibility Criteria and Study Selection 

We included observational studies, defined as case–control studies and cohorts, conducted on humans and written in the English language. Animal studies were excluded. We excluded papers that did not fulfill the aims of our study. After screening the studies, we included papers with one of the following characteristics: (1) patients with KLS; (2) intervention: use of functional imaging to evaluate the patients such as fMRI, SPECT; (3) comparator: there was not a comparison because of the low prevalence of the disease; and (4) outcomes: areas of the brain with hypo/hypermetabolism, hypo/hyperactivity, during episodes and between the episodes of KLS.

#### 2.1.2. Database and Search Strategy 

We used the PubMed and Google Scholar databases for this systematic review. The search was conducted between December 2021 and January 2022. We used an advanced search strategy with the following terms: (“Kleine Levin Syndrome” [Title/Abstract] AND “MRI” [Title/Abstract]) OR (“Kleine Levin Syndrome” [Title/Abstract] AND “SPECT” [Title/Abstract]) OR (“Kleine Levin Syndrome” [Title/Abstract] AND “fMRI” [Title/Abstract]) OR (“Kleine Levin Syndrome” [Title/Abstract] AND “Imaging” [Title/Abstract]).

#### 2.1.3. Data Extraction and Analysis 

We collected from each paper in the first table the following information: the author, year, country, age, sex, number of participants, and methods of the study.

We collected from each paper in the second table the following information: the author, year, country, functional study, and areas with hyper/hypometabolism or hypo/hyperactivity. 

#### 2.1.4. Bias Assessment 

We used the Robins-1 analysis for bias assessment in each study [12]. 

## 3. Results

### 3.1. Figures, Tables, and Schemes

**Figure 1:**
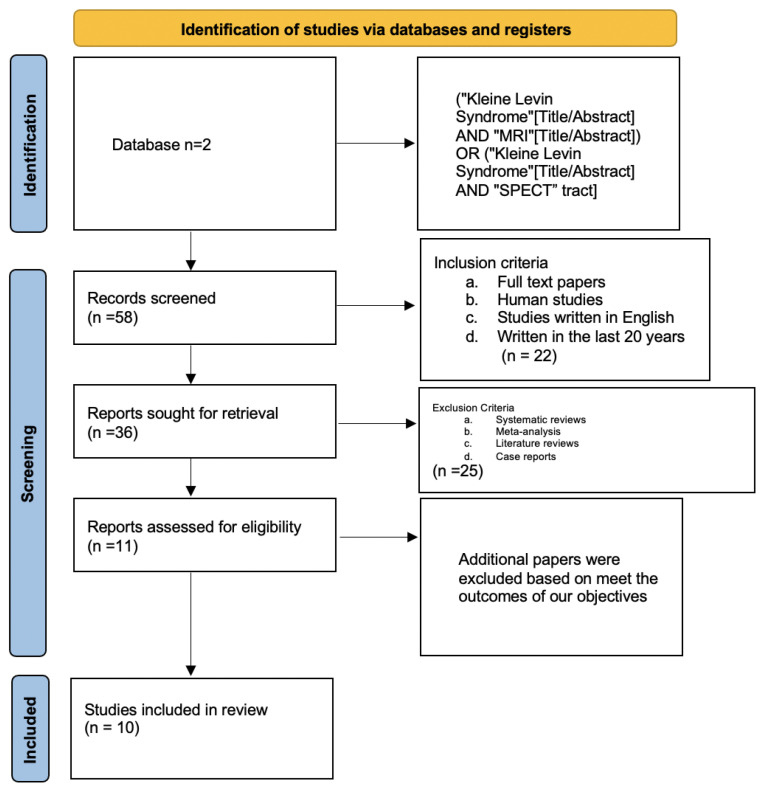
The PRISMA flow chart.

Figure 1 shows the PRISMA flow chart of the study.

### 3.2. Study Characteristics

We found 10 observational studies that specifically discussed the role of functional imaging in patients with K.L.S.; Table 2 shows the main characteristics of the studies [13,14,15,16,17,18,19,20,21,22].

### 3.3. Study Outcomes

There were five studies that reported cases in the asymptomatic period, one study in the symptomatic period, and four studies in both periods. Hypometabolism, less activity or hypoperfusion were found in seven studies such as Engstrom et al. in 2016, Engstrom et al. in 2009, Huang et al. in 2005, Dauvilliers et al. in 2014, Dudoignon et al. in 2021, Ket et al. in 2014, Engstet et al. in 2014. This hypometabolism was located mainly in the frontal, temporal, occipital, lobe, thalami, and cingulate zone. On the other hand, hypermetabolism, greater activity or hyperperfusion was established in five studies: Engstrom et al. in 2009, Dauvilliers et al. in 2014, Dudoignon et al. in 2021, Engstrom et al. in 2014, Vigren et al. in 2013. The increased of activity was presented mostly in the thalamus and prefrontal cortex. Table 3 shows the main findings in each study [13,14,15,16,17,18,19,20,21,22].

### 3.4. Bias Analysis

A total of 4 out of the 10 studies established limitations; a major limitation was the absence of a matched control group in Vigren et al., 2014 and Dudoignon et al., 2021. A heterogeneous group of patients participated; they had no similarities in state of disease, some were assessed while having K.L.S. episodes, while, others were in remission. Symptomatology and disease duration were not similar either. Due to these features, perfusion patterns could change and be associated with specific symptom patterns such as those found in Vigren et al., 2014. Additionally, in Dudoignon et al., 2021, the FDG-PET/CT was performed in a single expert center that is highly experienced in K.L.S., which means they were more capable of detecting subtle changes in K.L.S. brain imaging, unlike many other studies. Another limitation was found in Engstrom et al., 2014, where there was no adjustment for the time when the patients had their last episode in the data analysis. Finally, Vigren et al., 2013 includes some limitations, such as technical issues in Magnetic Resonance Spectography (M.R.S). Technology at the time of the study design. Additionally, the latter presented different limitations related to the analysis of the results, as the use of a voxel of interest in the M.R.S. study—including the entire thalamus and not only the anterior-medial parts—may also cause individual variation of N acetyl aspartic acid (NAA)-concentrations in both groups and could contribute to a variable distribution of the thalamic neurons. Furthermore, the absence of a significant difference in thalamic levels of N.A.A. between patients and controls is an argument against a primary thalamic dysfunction. Table 4 shows the bias analysis of the study [14,15,17,18,19,20,22,23].

## 4. Discussion

We found multiple relationships between K.L.S. clinical features and the different affected areas of the brain with different neuroimaging studies. Table 5 shows the different types of neuroimaging methods that were used in the studies for this systematic review. Table 5 shows the explanation of the imaging studies use in this publication [24,25,26].

### 4.1. Asymptomatic Period 

The asymptomatic period in K.L.S. is free of hypersomnolence attacks but working memory task deficits remain. Additionally, functional imaging is abnormal between episodes [13]. 

#### 4.1.1. Working Memory

Functional imaging in patients with K.L.S. is similar to other central parasomnias such as narcolepsy. Patients with narcolepsy have decreased activity in the frontal, anterior cingulate, and parietal areas. At the same time, the parietal region of patients with K.L.S. seems to be unaffected [23,27]. Additionally, increased thalamic activation and hippocampal activation correlated with working memory deficits in patients with K.L.S. [18].

#### 4.1.2. Thalamic Activation and Hippocampal Activation 

Three studies showed increased thalamic activity during the asymptomatic period, correlating with working memory task deficits using fMRI [15,22,23]. Hyperactivity of the thalamus during the symptomatic period could be explained as a compensatory mechanism [23]. Both studies were related to decreased working memory tasks, which contrasts with the symptomatic period where hypometabolism is found [13].

In the first two studies, thalamic hyperactivity correlated with lower activation in the anterior cingulate and dorsomedial prefrontal cortex [23]. The author suggests that the anterior cingulate and prefrontal deficits could be a consequence of thalamic pathology [23]. In contrast to these studies, Kas et al., using SPECT imaging, showed decreased perfusion bilaterally in the thalamus.

In Dudoignon et al.’s study, more patients with hippocampal hypometabolism had working memory deficits than those without hippocampal metabolism [19]. Executive functions, attention, and episodic verbal memory were preserved, suggesting that other areas of the brain compensate for the hypometabolism of the hypothalamus [19]. 

#### 4.1.3. Thalamic Activation and N.A.A. Levels

N-acetyl aspartate (NAA) is a biomarker of neuronal loss and malfunction, so a decrease in the NAA levels suggests neuronal deficit [15]. Vigren et al. showed a negative correlation between thalamic activity and NAA levels, and patients with high activity of the left thalamus period had low NAA levels between episodes. This negative correlation was statistically significant and not found in the control group while testing working memory tasks [15]. Nevertheless, NAA levels were not significantly different from controls [15].

#### 4.1.4. Frontal Eye Fields and Pons

Significant connections between the pons and the frontal eye field have been established before. In 2016 Engstrom et al. found decreased connectivity between the frontal eye fields and the pons compared to controls [20]. The frontal eye field has been linked to visual attention, which is associated with working memory [28], while nuclei adjacent to the pontine reticular formation have been reported to perform a role in sleep regulation, wakefulness, and oculomotor control [20].

A previous case report found a disrupted connection between the thalamus and the pons [29]. Still, the same study found normal connectivity between the thalamus and the pons compared to the control [29]. The existing hypotheses of the role of connectivity between the FEF and the pons merit further attention from researchers [20]. Significant connections between the pons and the frontal eye field have been established. In 2016, Engstrom et al. found decreased connectivity between the frontal eye fields and the pons compared to controls [20]. The frontal eye field has been linked to visual attention, which is associated with working memory [28]. At the same time, nuclei adjacent to the pontine reticular formation have been reported to perform a role in sleep regulation, wakefulness, and oculomotor control [20]. In the same study, there was normal connectivity between the thalamus and the pons as compared to the control; this was explored because of a previous case report that found this dysfunction [29]. The existing hypotheses of the role of connectivity between the FEF and the pons merit further attention from researchers [20].

#### 4.1.5. The Executive and Salient Networks 

The study by Engstrom 2014 analyzed working memory pathways in patients with K.L.S., specifically the executive network and the salience networks during the asymptomatic phase [16]. The salient network refers to the interaction areas to decide what merits our attention [30]. The executive network maintains and manipulates information in working memory and is responsible for problem-solving and decision-making [31].

Regarding the executive network, K.L.S. patients had increased activation in the left dorsolateral prefrontal cortex (DLPFC) and the left hemisphere in the region of the posterior parietal cortex (PPC) tested [16]. This finding is usually correlated with lower performance levels and longer reaction times, when working memory is tested [16].

Regarding the salience network, K.L.S. patients have increased activation of the left thalamus, more activation of the right anterior cingulate cortex (ACC), and decreased activation left insular cortex (AIC) [16]. The longer the syndrome duration, the more pronounced hypoperfusion during the asymptomatic period [13].

#### 4.1.6. Depersonalization/Derealization

Decreased perfusion in the temporal and occipital cortex was related to depersonalization and derealization. The more the patients suffered from depersonalization/derealization, the greater the affected area [18]. The correlation in the symptomatic period was more robust than in the asymptomatic period in the same study [18].

#### 4.1.7. Initial Markers

In the Dudoignon study, hypometabolism in the posterior associative cortex and hippocampus and increased metabolism in the prefrontal cortex was associated with the younger onset of age and shorter course of the disease [19]. Hypometabolism in the posterior associative cortex was also associated with a shorter course of the disease, and the association with the female sex was strong. Clinically, females have less hypersexuality, more depression, and a shorter course of disease as compared men. Hypometabolism in the hippocampus was also associated with birth problems [19].

### 4.2. Symptomatic Periods

#### 4.2.1. Hypersomnolence

Decreased level of consciousness can be explained by the involvement of the reticular activating system because there might be disruption between the connection in the thalamus (paramedial) and cerebral cortex. Lesions of the thalamic nuclei also have been reported to cause limbic symptoms [32]. Another example is patients with Percheron artery stroke, who present with ischemia of the bilateral paramedian thalamus. These patients show similar signs to K.L.S. patients, such as sleepiness, coma, memory impairment, hyperphagia, and apathy [33]. 

Other studies suggest that the connections between the brainstem and the thalamus can be diminished during a hyper somnolent state. This finding suggests a relationship between the thalamus and the brainstem, which regulate sleep and wakefulness [29]. The ascending arousal system consists of two pathways: a dorsal pathway from the upper brainstem through the hypothalamus and a dorsal path passing through the thalamus [29].

While imaging modalities have revealed hypoperfusion of both thalami on SPECT imaging during the symptomatic period in K.L.S. patients, its potential clinical implications have not been thoroughly investigated [13]. In contrast, Dauvilliers et al. also found increased activity in the left thalamus compared to controls [21]. However, there were only four individuals in this study, and a different study imaging was used (PET SCAN) [21].

#### 4.2.2. Depersonalization and Derealization

Depersonalization and derealization were correlated with hypoperfusion in the left and right parietal-temporal junction [18]. The angular gyrus located in Brodmann area 39 is involved in the complex modal association between auditory, visual, and somatosensory information. Patients with depersonalization disorders have been found to have abnormalities in the posterior part of Brodmann’s area 21, 22, and 39. The parietal-temporal function conveys the ability to embody ourselves alone and in relation to the movement of surrounding people [18].

#### 4.2.3. Apathy

There was more hypoperfusion as compared to control in the right dorsomedial prefrontal cortex [18]. Patients also tend to present with apathy (quantitative reduction in voluntary and purposeful behavior) [17]. Apathy could be explained by defects in the prefrontal and orbitofrontal cortex [18]. Patients with K.L.S. have more hypoperfusion than control in the right dorsomedial prefrontal cortex [18]. 

#### 4.2.4. Hypersexuality

As suggested by AlShareef et al., abnormal sexual behavior may orientate towards hypothalamic dysfunction; however, the hypothalamus seems to be intact [7]. This suggests that the anatomical structure related to the abnormally increased sex drive should reside somewhere else. Miglis et al. suggests that the behavioral symptoms observed in K.L.S., including hypersexuality, could be related to the orbitofrontal and anterior parasagittal abnormalities observed during symptomatic periods [34]. The striatum could have also a correlation with hypersexuality in patients with K.L.S., but there is not a clear neuroimaging correlation [14]. 

#### 4.2.5. Striatum Role and Additional Findings

There have been many reports showing the hypermetabolism of the striatum during the symptomatic period of patients with K.L.S. Some authors, such as Haba-Rubio et al., explain that this enhanced activity in the striatum occurs due to compensatory mechanisms surrounding the primarily affected regions [35]; however, Drouet et al. hypothesize that this activation reflects an implication of the thalamostriatal structures in the sleep and wakefulness regulation [36]. Hoexter et al. assessed the Dopamine Transporter (D.A.T.) availability in the symptomatic vs. asymptomatic phase of K.L.S., finding a marked reduction in the symptomatic phase, suggesting a dopaminergic dysfunction in this phase of K.L.S. [37]. However, more studies are required to establish the role between the Striatum and K.L.S. Additionally, Dauvilliers et al. results showed higher metabolism during the symptomatic episodes in paracentral, precentral, and postcentral areas, supplementary motor area, superior medial frontal gyrus, and bilaterally in the left thalamus and putamen [21]. 

### 4.3. Disease Progression and Remission

The disease seems to improve over time. In one study, there was less activation in the asymptomatic period in the anterior cingulate cortex, left precuneus, and left occipital cortex in the second examination compared to the first examination using fMRI [29]. A second study showed increased perfusion since the last visit in the orbitofrontal cortex. Additionally, there was decreased perfusion in the right patient-temporal cortex with a longer disease course [15]. Another study shows a minor degree of activation in the asymptomatic period in the anterior cingulate cortex, left precuneus, and left occipital cortex in the second examination as compared to the first examination which shows that the disease improves over time fMRI [29]. 

Regarding remission, almost half of the patients that have undergone remission showed the persistence of the same pattern. This may imply that some patients could have chronic and persistent cognitive deficits and maybe a non-basic course of the disease [16]. KLS patients tend to have working memory deficits after remission, which correlates with this finding [38]. 

### 4.4. Study Type 

Virgen et al. concluded that SPECT may be a diagnostic aid only for experienced neurologists able to recognize and consider K.L.S. and its differential diagnoses as potential causes of hypersomnia in young patients. SPECT has a low sensitivity (48%) and should not be used as the only diagnostic tool for K.L.S. [17]. In the Kas Study, thalamic activation is different from the results of other fMRI studies, so researchers and physicians should consider which type of imaging is used when conclusions are made. There is also a contradiction in Dauviller’s study, which showed increased thalamic perfusion instead of decreased perfusion in the thalamus. It is fair to point out that Dauviller’s study used P.E.T. instead of fMRI used in other studies [21]. 

## 5. Overview

**Figure 2:**
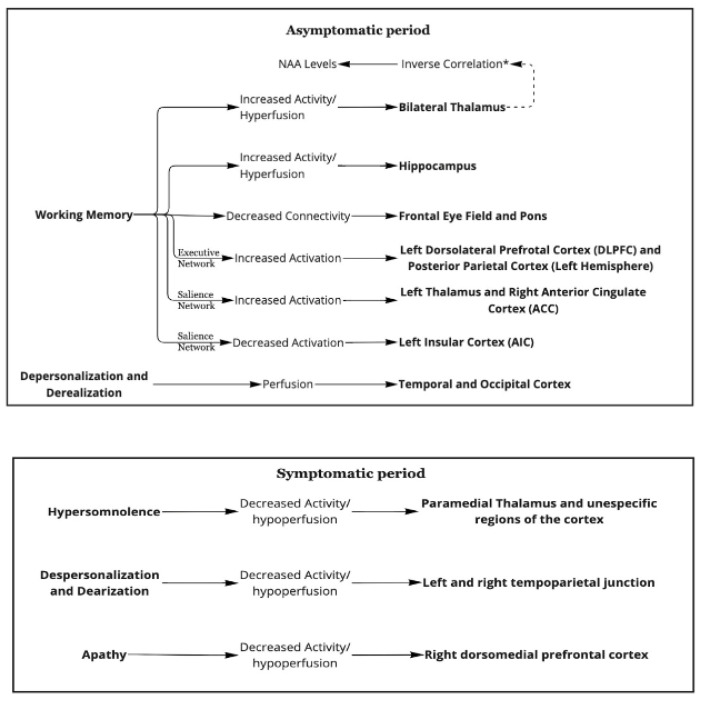
A graphic summary of the paper. * Greater activity in the thalamus is correlated with lower NAA levels.

Figure 2 shows the summary of the pathophysiology of K.L.S. based on neuroimaging studies [14,15,16,17,18,19,21,23,29].

## 6. Conclusions

Our findings reflect that there are changes in functional imaging studies during symptomatic, asymptomatic, and between episodes in patients with K.L.S. These changes vary according to the study (SPECT, PET SCAN, or fMRI) used to identify these modifications; some of them are conflicting.

Hyper insomnia, the main feature of this disease during the symptomatic periods, was associated with decreased thalamic activity. Other features of K.L.S., such as apathy, hypersexuality, and depersonalization, were also correlated with functional imaging changes. Some findings correlated with working memory deficits seen in this stage during the asymptomatic periods. Hyperactivity of the thalamus and hypothalamus were the main features shown with these working memory deficits during the asymptomatic periods. Additionally, functional imaging tends to improve with a longer course of the disease, suggesting that K.L.S. patients outgrow the disease. These findings should caution physicians when analyzing and correlating neuroimaging findings with the disease.

## Figures and Tables

**Table 1:** ICSD-3 criteria for Kleine–Levin Syndrome.

A ≥2 recurrent episodes of hypersomnia, each persisting for 2 days to 5 weeks B Episodes recur at least 1 every 18 months C There is normal sleep, cognition, behavior, and mood between episodes In addition, the patient should have at least one of the following: (1) Hyperphagia (2) Hypersexuality (3) Abnormal behavior such as irritability, aggression (4) Cognitive dysfunction such as confusion, derealisation, hallucinations

**Table 2:** Main characteristics of the study.

Author, Year, Country	Number of Participants, (M/F), Mean Age	Age	Study Type/Single-Center/Multicenter	Methods
Huang et al., 2005, Taiwan [13]	7 K.L.S. patients (7 M, 0 F)	Participants: 13.4 years Control: No cases	Cross-Sectional	SPECT studies were conducted in the asymptomatic period in all the patients (*n* = 7). Studies were conducted in the symptomatic period only in 5 patients. There was no control group.
Engstrom et al., 2009, England [23]	8 participants (5 M, 3 W), 12 controls	Participant: 27 years, Controls: 24.1	Cross-Sectional/Single Center.	(fMRI) applying a verbal working memory task was used in conjunction with a paper-and-pencil version of the task. Seven patients had an active disease, and one was in remission. All patients were asymptomatic at the time of the fMRI.
Vigren et al., 2013, Sweden [15]	14 K.L.S. patients, 15 healthy controls	Participants: 14.7 Controls: 22.1	Cross-sectional, Single-center	Patients diagnosed with KLS. according to ICSD were enrolled. All controls were recruited after a paper-and-pencil version of a reading-span task by Daneman and Carpenter.
Engström et al., 2013, England [16]	44 participants (24 F, 20 M) 26 controls, 18 KLS patients	Participant: 24.1 years Control: 24.7 years	Cross-Sectional	Working Memory was assesed by using FMR. The participants of the study were divided in low capacity and high capacity groups according to a performance of memory task.
Kas et al., 2014, France [18]	41 K.L.S. patients, 15 healthy control	Participants: 22.3	Cross-sectional, Single-center Study	A total of 70 patients with primary K.L.S. diagnosis were enrolled after removing 35 suspected cases referred to the center.
Vigren et al., 2014, Sweeden [17]	24 K.L.S. patients, no controls	Not reported	Cross-sectional, Single center Study	SPECT SCAN was used in all the patients in the symptomatic period. SPECT SCAN was also performed in the asymptomatic period in 21 patients. Patients were categorized as severe and non-severe.
Dudoignon et al., 2021, France [19]	138 K.L.S. patients, no control	21.6	Cross-Sectional Study, Single-center	The confirmed 210 K.L.S. patients were enrolled out of 260 suspected K.L.S. patients referred to the center after a cognitive assessment, blood sampling, and an interview with K.L.S. physicians.
Engstrom et al., 2014, Sweden [22]	18 K.L.S. patients, 26 healthy controls	Participants: 25.9 Controls: 24.1	Cross-sectional study, Single center	According to the International Classification of Sleep Disorders, 18 patients diagnosed with K.L.S. were included. The healthy controls were recruited after a thorough evaluation by a clinical interview.
Engstrom et al., 2016, England [20]	12 Participants (4 M, 8 F), 14 controls,	Participant: 23.8 years (SD = 9.1 years). Controls: 24.1	Cross Sectional Study/Single Center	All participants were asymptomatic at the time of the study. The participants of the study were matched to controls.
Dauvilliers et al., 2014, France [21]	15 healthy control 4 K.L.S.	participants: 16.25 years Control: 28	Cross-sectional, Single-center Study	Four K.L.S. patients underwent F-FDG-PET scanning from day 2 to day 3 after the symptomatic episode and two to three months after the last day of the symptomatic episode. Fifteen controls were included for comparison.

**Table 3:** The main findings of each study.

Author, Year, Country	Imaging	Hypometabolism/Less Activity/Hypoperfusion	Hypermetabolism/Greater Activity/Hyperfusion
Huang et al., 2005, Taiwan [13]	SPECT with 925 MBq (25 mCi) of technetium-99 m ethyl cysteinate dimer (Tc-99 m ECD)	All the patients had hypoperfusion of both thalami during the symptomatic period. In the symptomatic and asymptomatic period, there was hypoperfusion in the temporal lobe, frontal lobe, and basal ganglia.	
Engstrom et al., 2009, England [23]	fMRI—BOLD response 1.5 T body scanner—in the asymptomatic period.	Reduced frontal activity in the anterior cingulate and prefrontal cortex while performing a reading span task.	Increased thalamic activity while performing reading and span tasks.
Vigren et al., 2013, Sweden [15]	fMRI 1.5 T in the asymptomatic period	There was a negative correlation between activity in the thalamus and N.A.A. levels.	Decreased N.A.A. levels when there was high activity in the left thalamus. High activity in the left thalamus while performing W.M. task. Larger activation in bilateral parietal cortex compared to controls.
Engstrom et al., England, 2013 [16]	fMRI-BOLD- 1.5 T body scanner	Salient network: decreased activation of the left insular cortex (A.I.C.).	Salient network: Increased activation of the left thalamus, more activation of the right anterior cingulate cortex (A.C.C.) Executive network, K.L.S. patients had increased activation in the left dorsolateral prefrontal cortex (DLPFC) and increased left hemisphere activation in the region of the posterior parietal cortex (P.P.C.) tested.
Kas et al., 2014, France [18]	SPECT Tc-99 m ECD- in the symptomatic and asymptomatic phase	Compared to control, K.L.S. patients had hypoperfusion in the hypothalamus, the thalamus, mainly the right posterior part, the caudate nucleus, and cortical associative areas including the anterior cingulate, the orbitofrontal, and the right superior temporal cortices during the asymptomatic period, while hypoperfusion in the right dorsomedial prefrontal cortex and the right parietal-temporal junction was noted during the symptomatic period.	Depersonalization/derealization- temporal-occipital relation, r = −0.79.5, *p* = 0.01) in the asymtomatic period. Depersonalization/derealization- temporal-occipital relation, r = −0.45, *p* = 0.05) in the asymtomatic period. The perfusion during the asymptomatic period in the right parieto-temporal r = 0.53, *p* = 0.05 decreased with each episode.
Vigren et al., 2014, Sweden [17]	SPECT with 650 MBq 99 m-Tc-HMPAO	A total of 48% have abnormal perfusion. Severe patients: 5/13 had temporal and/or frontal hypoperfusion. Non-severe patients: 7/12 had temporal and/or frontal hypoperfusion. Patients with active disease: 7/16 had temporal and/or frontal hypoperfusion. Patients with remission: 5/9 had temporal and/or frontal hypoperfusion.	
Dudoignon et al., 2021, France [19]	FDG-PET FDG-PET/CT using Gemini Dual PET/CT 30 min post- injection of 2 MBq/kg FDG -in the asymptomatic period	A total of 70% of 138 had hypometabolism in the left temporo-occipital junction A total of 63% hypometabolism bilaterally posterior associative cortex A total of 50% have the entire homolateral, bilateral posterior associative cortex, and hippocampus.	Prefrontal, dorsolateral cortex was noted in 34.8% of patients, more often on the right than the left side.
Engstrom 2014 [22]	fMRI 1.5 T- in the asymptomatic period in the asymptomatic state	K.L.S. patients illustrated reduced activation in the medial frontal and anterior cingulate cortices during (*p* < 0.001).	Increased thalamic activation in 61.4% of patients.
Engstrom et al., 2016, England [20]	fMRI/SPECT in asymptomatic patients	Patients with Kleine–Levin syndrome showed less activity in between the pons and the frontal eye fields as compared to controls at the asymptomatic period (*p* = 0.041).	
Dauvillers et al., 2014, France [21]	PET with F-fluorodeoxy glucose (F-FDG)	K.L.S. patients exhibited hypometabolism in occipital and temporal gyri and in the inferior parietal areas compared to control during the symptomatic phase.	As compared to healthy individuals, the 4 K.L.S. patients demonstrated hypermetabolism in paracentral, precentral, postcentral areas, medial frontal gyrus, thalamus, and putamen during symptomatic periods. In the asymptomatic phase, the 4 K.L.S. patients revealed having more hypermetabolism in frontal and temporal cortices, posterior cingulate, and precuneus as compared to controls.

**Table 4:** The bias analysis of the systematic review.

Author, Year	Confounding	Selection of Participants	Classification	Deviations	Missing Data	Measurements	Selection of Reported Results
Dudoignon [19]	Medium risk	Moderate risk	Low risk	Low risk	Moderate risk	Low risk	Low risk
Kas et al., 2014 [18]	Low risk	High risk	Low risk	Low risk	Low risk	Low risk	Low risk
Dauvillers et al., 2013 [21]	Low risk	Moderate risk	Low risk	Low risk	Low risk	Moderate risk	Low risk
Engstrom et al., 2013 [16]	Low risk	Moderate risk	Low risk	Low risk	Low risk	Moderate risk	Low risk
Ensgtrom et al., 2016 [20]	Low risk	Low risk	Moderate risk	Low risk	Low risk	Moderate risk	Low risk
Engstrom et al., 2014 [22]	Low risk	Moderate risk	Low risk	Low risk	Low risk	Low risk	Low risk
Engstrom et al., 2009 [23]	Low risk	High risk	Low risk	Low risk	Moderate risk	Low risk	Moderate risk
Vigren et al., 2013 [15]	Low risk	Moderate risk	Low risk	Low risk	Low risk	Moderate risk	Low risk
Vigren et al., 2014 [17]	Low risk	High risk	Low risk	Low risk	Moderate risk	Low risk	Low risk

**Table 5:** Explanation of Imaging studies.

Imaging Study	How It Works
Single-photon emission C.T. (SPECT) [24]	It creates a 3D image by the representation of a radioactive tracer (e.g., technetium-99 m) inserted in the body, allowing the identification of functionality and perfusion of different tissues, in this case, the brain.
Functional M.R.I. (fMRI) [25]	It measures hemodynamic response induced by neuronal activity and measured though a blood oxygen level dependent (BOLD) signal which depends on oxy/deoxy haemoglobin concentration.
Fluorodeoxyglucose positron emission tomography (FDG-PET) [26]	F.D.G. is a glucose analog metabolized by tissues with a high glucose demand (e.g., cancers, heart, and brain) and is measured using a tracer. In that way, this study allows us to identify brain activity by measuring the uptake of F.D.G. and indirectly measuring the blood flow through the brain.

## Data Availability

Not applicable.
